# Generative Ai for Cardiovascular Cell Type-Specific Fluorescence Colorization of Live-Cell hPSC-Derived Cardiac Organoids

**DOI:** 10.1002/aidi.202400041

**Published:** 2025-04-24

**Authors:** Arun Kumar Reddy Kandula, Tanakit Phamornratanakun, Angello Huerta Gomez, Marcel El-Mokahal, Zhen Ma, Yunhe Feng, Huaxiao Yang

**Affiliations:** 1 Department of Biomedical Engineering, University of North Texas, Denton, Texas, USA; 2 Department of Computer Science & Engineering, University of North Texas, Denton, Texas, USA; 3 Department of Biomedical & Chemical Engineering, Syracuse University, Syracuse, New York, USA; 4 BioInspired Syracuse Institute for Material and Living Systems, Syracuse University, Syracuse, New York, USA

**Keywords:** bright-field microscope, cardiac organoids, fluorescence imaging, generative adversarial networks, human pluripotent stem cell (hPSC), image colorization

## Abstract

Human pluripotent stem cell (hPSC)-derived cardiac organoids (COs) are the most recent three-dimensional tissue structure that mimics the human heart’s structure and functionality for modeling heart development and disease. Fluorescent labeling and imaging are commonly utilized to characterize the cellular information in COs. However, the additional step of fluorescence labeling and imaging is time-consuming, inefficient, and typically for end-timepoint characterization. Meanwhile, the COs are routinely examined by brightfield/phase contrast microscope to track live-cell organoid formation in structure and morphology. Although the brightfield microscope provides essential information about COs, such as morphology and overall structure, it limits our understanding of cardiovascular cells (e.g., cardiomyocytes, CMs and endothelial cells, ECs) and corresponding quantifications in COs. Is it possible to overcome these limitations of bright-field microscopic imaging and provide cardiovascular cell type-specific information similar to the fluorescence-labeled imaging acquisition in COs? This research addresses this limitation by proposing a generative AI system for colorizing phase contrast images of COs from bright-field microscopic imaging using conditional generative adversarial networks (cGANs) to generate cardiovascular cell type-specific fluorescence images of COs. By giving these phase contrast images with multichannel fluorescence colorization, this intelligence system unlocks cell type and quantifications of COs in high efficiency and accuracy.

## 1 | Introduction

Human pluripotent stem cell (hPSC)-derived cardiac organoids (COs) have been developed exclusively and marvelously for modeling cardiovascular development and disease in various biomedical applications of regenerative medicine and pharmaceutical discoveries [1–5]. To routinely monitor CO differentiation and morphology, bright-field/phase contrast microscopic imaging has been conveniently and significantly utilized in nearly all labs for live cell and organoid culture and characterization [6–8]. However, the bright-field/phase contrast microscopic imaging is not sufficient for effectively understanding COs at the cellular level, specifically cardiovascular-specific cells, including cardiomyocytes (CMs) and endothelial cells (ECs) [8, 9]. To take advantage of the transgenic hPSC line encoding cell type-specific promoters with fluorescence reporters, others and we labeled and tracked the process of hPSC differentiation into the cardiovascular cells by live-cell fluorescence microscopic imaging [10–12]. Since each fluorescence signal corresponds to a specific cell type and corresponding cellular quantification, the transgenic reporter hPSC lines have been extremely helpful in tracking temporospatial CO formation at the multicellular level to extend the application of COs in heart-relevant disease modeling and drug screening by fluorescence microscopic imaging. However, fluorescence microscopic imaging of different cell types in the hundreds of COs is time-consuming and has low throughput. Is it possible to label major cardiovascular cell types with designated colors (also known as image colorization) on the brightfield/phase contrast images of COs without fluorescence staining or labeling?

Artificial intelligence (AI) has been extensively explored to tackle the challenge of image colorization. Traditional machine learning (ML) approaches rely on extracting similar features from a reference image to predict the colors in a new image [13–15]. However, the performance of these methods is heavily dependent on the degree of similarity between the reference image and the target. The advent of deep learning (DL) techniques, such as convolutional neural networks (CNNs), marked a significant shift, allowing for the automatic extraction of features from images. Pretrained CNNs have gained prominence in image colorization, leveraging feature maps to predict pixel colors [16,–21]. The capabilities of generative adversarial networks (GANs) [22] in various generative tasks have prompted their use in colorization. In this context, conditional GANs, exemplified by Pix2Pix GAN [23], have emerged, mapping grayscale inputs to corresponding ground truth images. Despite the technological advancement of image colorization on generic image categories, there is a lack of research focused on colorizing in vitro cell culturing systems, specifically the hPSC-derived organoids. Typically, small color discrepancies are tolerable for generic image generation, while it might be detrimental to COs’ images containing much smaller features to introduce significant misinformation and further render the task of organoid colorization exceptionally challenging. Recently, GAN-based methods for generating fluorescence in COs have been implemented. In one approach, 2D CO images were converted into 2.5D representations by incorporating morphological information and employing cGANs along with image segmentation to predict the regions where fluorescence should be present. These regions were then filled with the corresponding fluorescence [24]. Despite this additional morphological guidance, the reported structural similarity index (SSIM) scores for these methods are low.

Therefore, we have established a novel framework utilizing cGANs with adversarial training between the generator and discriminator [23] for training on CO images (phase contrast and corresponding fluorescence images in GFP-CM and mOrange-EC) with an attention mechanism of convolutional block attention module (CBAM) [25], to ensure an increased emphasis on small details and generate more accurate colors. Moreover, the predicted colorized images were evaluated by the three well-accepted evaluation metrics: peak signal-to-noise ratio (PSNR), SSIM, and weighted patch histogram (WPH). The image quantifications of cell type-specific fluorescence area and intensity in COs were also applied to compare with the ground truth. Collectively, we established a novel AI platform for colorizing and quantifying the fluorescence images of CMs and ECs in COs from the live-cell brightfield/phase contrast images.

## 2 | Experimental Section

### 2.1 | hPSC-Derived CO Differentiation and Microscopic Imaging

The COs were obtained based on our previous protocolfor directly differentiating vascularized COs from the hPSCs (Stanford Cardiovascular Institute Biobank, SCVI 3R), which were micropatterned by a stencil at a size of 2000 μm circle. Through the treatment of CHIR-99021 from day 0 to 2 and IWR-1 from day 3 to 5, and then combined growth factors and small molecules of 50 ng/mL VEGF (VEGF) (100-20, PeproTech); 5 ng/mL FGF-2 (FGF2) (100-18B, PeproTech); 10 μM SB431542 (SB) (S1067, Selleck Chemicals); 50 ng/mL Angiopoietin-2 (ANG2) (130–07, PeproTech); 50 ng/mL Angiopoietin-1 (ANG1) (130–06, PeproTech) from day 5 to 16 for cardiac vascularization, the myocardial ring-like COs were obtained at day 16. Since the hPSC line was transgenically encoded with fluorescence reporters [12], we were able to image the COs with live-cell bright-field/phase contrast and fluorescent microscopy (Keyence All-in-One Fluorescence Microscope BZ-X810 equipped with a highly sensitive and Peltier cooling/5°C CCD camera) in Green (G)–GFP–TNNT2–CM (GFP labeled TNNT2 promotor for CMs) and Red (R)–mOrange–CDH5–EC (mOrange labeled CDH5 promotor for ECs) and corresponding phase contrast. The CCD camera is 2/3 inch, 2.83 M pixel monochrome, and colored by a LC filter. The Keyence Imaging System adapts the transmitted light source with a 3.7 W LED and fluorescent light source with a 40 W LED. A consistent imaging setting of every batch of CO image is summarized below:

**Table:** 

	Brightfield (oblique illumination ON)	GFP-EGFP/C206082 (filter for green)	RFP-ET-CY3/R/C206083 (filter for red)
Excitation light	25%	100%	100%
Exposure time (s)	1/2800	1/8.5	1/8.5

A total of 1374 paired images (phase contrast and fluorescence) originally in RGB color mode were used for training, while 79 paired images, including new batches of CO differentiation, were used for testing and evaluation. All the CO images are 24-bit images resulting in each pixel having an intensity range of 0–255 for each channel in RGB color space. Conversion of the CO images to CIELAB color space is only happening during the training process for efficiently separating the grayscale and color information from the images; in this conversion, each pixel in Lightness channel will have a range of 0–100 and each pixel in a* and b* channels will be in a range of −128 to 127. For training efficiency, the pixels here are normalized to a range of −1 to 1 and rescaled back to the initial range after the fluorescence generation and saved as png images. In the evaluation phase and the quantization phase, the saved images were loaded in RGB color space and each pixel here had a standard range of 0–255 in each channel. To make the model better capture the intricate fluorescence details from a limited dataset, the majority of the data were allocated for training. This approach aims to maximize the model’s exposure to diverse examples and the remaining subset of data was used for testing and evaluation to assess the model’s performance.

### 2.2 | Framework Overview

The conditional GAN (cGAN) was built by utilizing the Pix2Pix model as the backbone [23]. The CIELAB color space was adopted to achieve this, consisting of three channels: Lightness, a*, and b*. In CIELAB, Lightness represents the grayscale channel, while a* and b* represent the two-color channels. This Lightness channel served as the conditional input to the generator, and the a* and b* channels were the target channels for generating colorized versions of the grayscale phase contrast images. The objective of using CIELAB color space was to extract only the color information from the COs and train the model to generate the plausible colors of a* and b* that were merged on the grayscale input to obtain the colorized images of COs. The conversion from the original images in RGB to CIELAB was established by the following two-step process of (a) converting RGB to *XYZ* and then (b) converting *XYZ* to CIELAB (L*, a*, b*):

#### 2.2.1 | Gamma Decoding

Since RGB values are gamma-encoded, they are first linearized using the piecewise RGB inverse gamma function. This step ‘undoes’ the gamma compression, resulting in linearized R, G, B values.

#### 2.2.2 | sRGB to XYZ

After linearization, the $\left[R_{\text{linear}},G_{\text{linear}},B_{\text{linear}}\right]$ vector was multiplied by the standard 3 × 3 RGB-to-*XYZ* matrix (for D65 white point):

$$\left[\begin{array}{l} X\\ Y\\ Z \end{array}\right]=\left[\begin{array}{lll} 0.4124 & 0.3576 & 0.1805\\ 0.2126 & 0.7152 & 0.0722\\ 0.0193 & 0.1192 & 0.9505 \end{array}\right]\left[\begin{array}{l} R_{\text{linear}}\\ G_{\text{linear}}\\ B_{\text{linear}} \end{array}\right]$$

#### 2.2.3 | XYZ to Lab

Then, the resulting *X*, *Y*, *Z* was normalized by the D65 reference white values $\left(X_{\text{n}},Y_{\text{n}},Z_{\text{n}}\right)$. From there, the standard Lab nonlinear transformations using the piecewise function was applied:

$$f(t)=\{\begin{array}{ll} t^{1/3} & t>\delta^{3}\\ \alpha t+\beta & \text{otherwise} \end{array}$$

with δ=6/29, leading to

$$L^{\text{*}}=116f\left(\frac{Y}{Y_{n}}\right)-16,\;a^{\text{*}}=500\left[f\left(\frac{X}{X_{n}}\right)-f\left(\frac{Y}{Y_{n}}\right)\right]\;b^{\text{*}}=200\left[f\left(\frac{Y}{Y_{n}}\right)-f\left(\frac{Z}{Z_{n}}\right)\right]$$

These Lab values serve as label inputs to the discriminator. Because Lab better reflects human perceptual uniformity, it offers more meaningful color-based differentiation within our training process. Standard libraries (e.g., OpenCV’s cv2.cvtColor or scikit-image) can perform this procedure internally, ensuring consistency and reproducibility.

**FIGURE 1 |:**
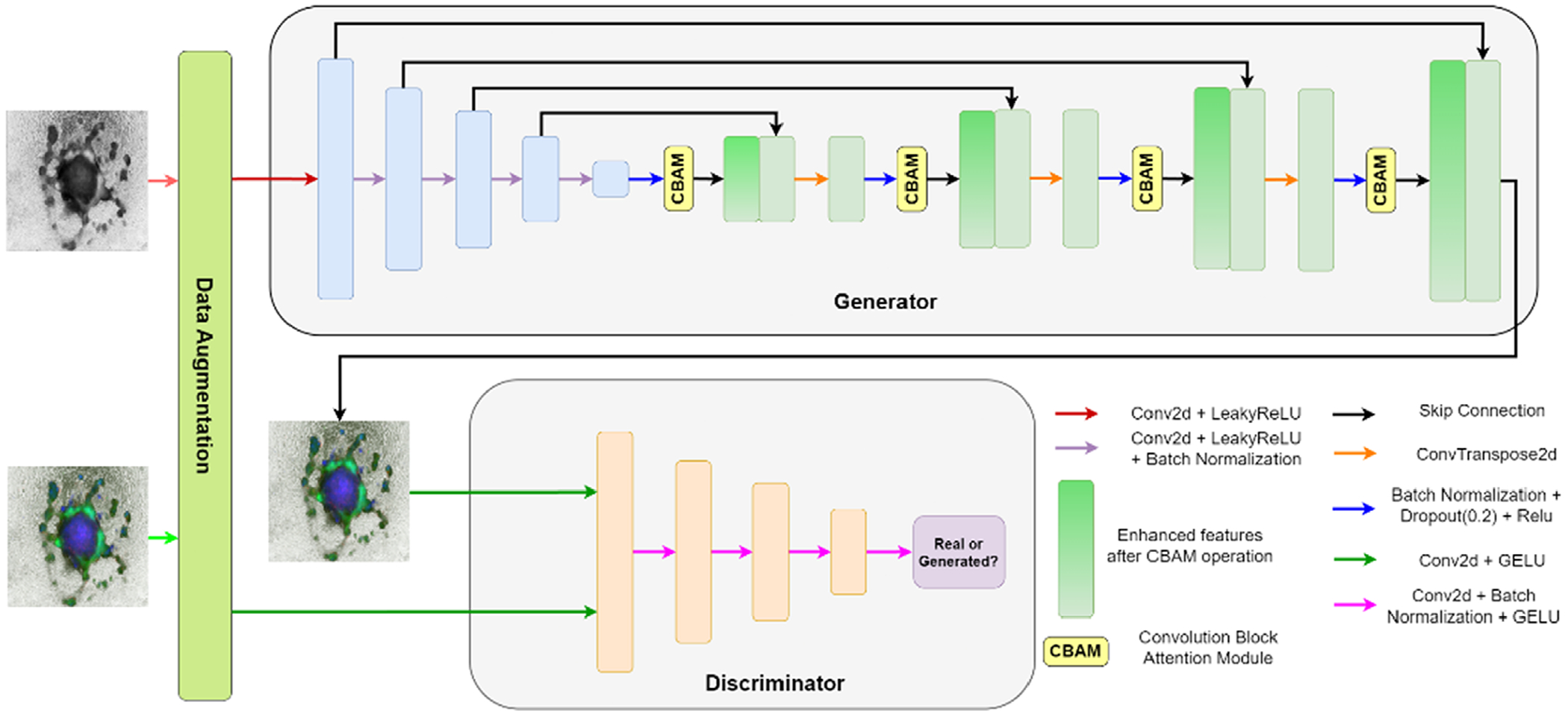
GAN architecture overview of fluorescence colorization of hPSC-derived COs.

Additionally, the CBAM [25] was incorporated to increase the channel and spatial attention of the GAN model to focus on the relevant features. CBAM is an innovative enhancement introduced to the architecture of deep neural networks, particularly CNNs. CBAM integrated both channel and spatial attention mechanisms, facilitating the model’s ability to focus on pertinent features within the input data. Channel attention enables the network to adaptively assign importance to different channels, emphasizing relevant information while suppressing noise. Simultaneously, spatial attention ensures that the network allocates its focus to meaningful spatial regions within an image. Figure 1 illustrates the main components and steps of the process of the image colorization workflow, which depicts the transformation of a grayscale CO image to a fully colorized output using Pix2Pix conditional GAN. The conditional input passed to the generator is the Lightness channel, and the discriminator was trained on the a* and b* channels.

## 3 | Individual Models

### 3.1 | U-Net Generator

The U-Net generator consists of an encoder and a decoder, connected by a bottleneck layer. Figure S1 demonstrates the architecture of our U-Net generator where the encoder progressively reduces the spatial dimensions of the input grayscale image while extracting features. The decoder then upsampled these features to produce the final colorized output. Skip connections between corresponding encoder and decoder layers facilitated the flow of low-level features, enhancing the network’s ability to capture fine details.

One distinctive feature of the U-Net generator here was its utilization of the Lightness (L) channel from the CIELAB color space as a conditional input. This L channel represents the grayscale information of the input image. By incorporating this channel, the generator focused on producing color information (a* and b* channels) that is coherent with the grayscale content.

### 3.2 | CBAM

The generator’s ability was enhanced using the CBAM, which integrates channel and spatial attention mechanisms, enabling the discriminator to adaptively assign importance to different channels and meaningful spatial regions within the image. CBAM (Figure S1) is an integral component incorporated into our U-Net generator architecture to enhance its ability to capture and emphasize relevant features within grayscale organoid images. The input feature map is $F\in R^{C\times H\times W}$ and CBAM extracts a 1D channel attention map $M_{c}\in R^{C\times 1\times 1}$ and a 2D spatial attention map $M_{s}\in R^{1\times H\times W}$. In summary, the overall attention processes can be explained as

(1) $$F^{\prime }=M_{\text{c}}(F)⊗F,F^{\prime \prime }=M_{\text{s}}\left(F^{\prime }\right)⊗F^{\prime }$$

where denotes the element-wise multiplication and the resulting $F^{\prime \prime }$ is the final refined output map that includes the details from both channel attention and spatial attention. This operation allowed the model to focus on relevant features while suppressing irrelevant information [25].

Channel attention enabled the network to adaptively assign importance to different channels of feature maps, emphasizing relevant information while suppressing noise. Channel attention is essential when dealing with multichannel images such as the L*a*b* color space we operate in. This selective channel weighting allows the model to focus on the most informative colorization components.

Spatial attention is another crucial aspect of CBAM. It ensures that the network allocates its focus to meaningful spatial regions within an image. In the context of colorization, this is especially important as it guides the model to concentrate on the relevant regions where colorization details are essential. Spatial attention complements channel attention by pinpointing critical areas in the input.

### 3.3 | Patch Discriminator

The patch discriminator is a CNN designed to operate on image patches rather than entire images as shown in Figure S2. This approach allows the discriminator to focus on local details and textures, making it well-suited for assessing the quality of colorizations at a fine-grained level. It consists of multiple convolutional layers to produce a single feature map that is used to classify the patch as real or fake. The final classification result for the entire image is obtained by averaging the predictions from the patches across the entire image. The result is a global classification score that represents the discriminator’s assessment of the overall image.

The patch discriminator engaged in adversarial training with the U-Net generator. It aims to distinguish between real colorized organoid patches and fake patches generated by the generator. Through this adversarial process, the discriminator provided feedback to the generator, encouraging it to produce colorizations that are indistinguishable from real color images.

The primary objective of the patch discriminator was to guide the U-Net generator in generating high-quality colorizations. Assessing the local realism of colorized patches helps ensure that fine-grained details and textures are faithfully preserved in the output.

### 3.4 | Loss Functions

#### 3.4.1 | Discriminator Loss

The discriminator, a key component of our conditional GAN, serves the crucial role of assessing the authenticity of colorized organoid images. To fulfill this role, the binary cross-entropy loss (BCEWithLogitsLoss) was used.

Mathematically, the discriminator loss can be expressed as

(2) $${ℒ}_{D}=-\frac{1}{N}\overset{N}{\underset{i=1}{\sum }}\left[y_{i}\cdot \text{log}\left(D\left(x_{i}\right)\right)+\left(1-y_{i}\right)\cdot \text{log}\left(1-D\left(G\left(L_{i}\right)\right)\right)\right]$$

Here, ${ℒ}_{D}$ represents the discriminator loss, where *N* is the batch size, $x_{i}$ denotes the ground truth colorized organoid images, and $y_{i}$ represents labels for real images ($y_{i}=1$) and fake images $\left(y_{i}=0\right)$. $D\left(x_{i}\right)$ signifies the discriminator’s output for real images, and $G\left(L_{i}\right)$ signifies the generator’s output for the corresponding grayscale input $\left(L_{i}\right)$. The BCEWithLogitsLoss computes the binary cross-entropy loss by comparing the discriminator’s predictions with the ground truth labels. To be specific, if $y_{i}=1$ (real sample), $\text{log}\left(D\left(x_{i}\right)\right)$ encourages the discriminator *D* to output a probability close to 1 for real inputs $x_{i}$. Taking the negative log pushes $D\left(x_{i}\right)$ to be large (close to 1). If $y_{i}=0$ (fake sample), $log\left(1-D\left(G\left(L_{i}\right)\right)\right)$ encourages *D* to output a probability close to 0 for generated inputs $G\left(L_{i}\right)$. Taking the negative log pushes $D\left(G\left(L_{i}\right)\right)$ to be small (close to 0).

#### 3.4.2 | Generator Loss

The generator, a pivotal component of our conditional GAN, was tasked with generating plausible colorizations. To achieve this, a combination of two loss functions, BCEWithLogitsLoss and L1 loss (mean absolute error), was used. Similar to the discriminator, BCEWithLogitsLoss as its adversarial loss function was used. It encourages the generator to produce colorizations that convincingly fool the discriminator into classifying them as real.

Mathematically, the generator’s adversarial loss is defined as

(3) $${ℒ}_{G_{GAN}}=-\frac{1}{N}\overset{N}{\underset{i=1}{\sum }}\text{log}\left(D\left(G\left(L_{i}\right)\right)\right)$$

This loss drove the generator to produce colorizations that were perceptually similar to real color images.

#### 3.4.3 | L1 Loss (Mean Absolute Error)

In addition to the adversarial loss, the L1 losswas incorporated to ensure that the generated colorizations closely match the ground truth images in terms of pixel-wise similarity.

Mathematically, the generator’s L1 loss is expressed as

(4) $${ℒ}_{G_{L1}}=\frac{1}{N}\overset{N}{\underset{i=1}{\sum }}{\left|G\left(L_{i}\right)-C_{i}\right|}_{1}$$

Here, $L_{G_{L1}}$ represents the generator’s L1 loss, where *N* is the batch size, $L_{i}$ denotes the grayscale input images, $G\left(L_{i}\right)$ represents the generator’s colorized output, and $C_{i}$ denotes the corresponding ground truth color images. The L1 loss encourages the generator to produce colorizations that closely match the ground truth, focusing on fine-grained pixel-level details.

By combining these two loss components ${ℒ}_{G_{\text{GAN}}}$ and ${ℒ}_{G_{L1}}$, the generator was trained to produce colorized organoid images that are both visually convincing and pixel-wise accurate, ultimately enhancing the quality and realism of the generated colorizations.

## 4 | Image Similarity Measurement Metrics

Evaluating the accuracy and quality of the generated image is a challenging task and on top of that, only a limited dataset of 1300 images was included. Therefore, non-deep-learning metrics were used to obtain a similarity score with three different evaluation metrics, PSNR, SSIM, and WPH, to compare the similarity between ground truth and colorized images.

PSNR was used to measure the quality of reconstructed or compressed images and this metric is used for comparing the similarity of the colorized image with ground truth [26–30]. It objectively measures how well a colorization technique preserves the details and visual fidelity of the original image. By calculating the PSNR value, the accuracy and fidelity of colorization algorithms were evaluated. Its range is (0, ∞), where 0 represents no similarity between images, and the higher the score the higher the similarity.

PSNR score of an m×n (width×height) image *I* and its compressed image *K* can be determined by

(5) $$20\cdot {\text{log}}_{10}\left({\text{MAX}}_{I}\right)-10\cdot {\text{log}}_{10}(\text{MSE})$$

where $\left({\text{MAX}}_{I}\right)$ is the maximum possible pixel value of the image and MSE is the mean square error of the original image *I* and its compressed image *K*; it can be calculated by

(6) $$\frac{1}{mn}\overset{m-1}{\underset{i=0}{\sum }}\overset{n-1}{\underset{j=0}{\sum }}{\left[I(i,j)-K(i,j)\right]}^{2}$$

The SSIM is a widely used evaluation metric for assessing the visual quality of the colorized image with ground truth [26–30]. It considers global and local image characteristics, capturing the perceptual differences and structural similarities between the colorized and ground truth images. To be specific, SSIM compares three components in an image pair, suppose *x* and *y* are the two patches of the true and compressed image, respectively, that are aligned with each other, the luminescence comparison function l(x,y) captures the differences in brightness, the contrast comparison function c(x,y) accesses variation in image contrast, and the structure comparison function s(x,y) measures differences in image structure and texture.SSIM is a combination of all these three factors [31].


(7) SSIM(x,y)=[l(x,y)⋅c(x,y)⋅s(x,y)]

By evaluating the preservation of underlying structures and textures, SSIM provides a comprehensive measure of the algorithm’s ability to maintain visual coherence and realism. The SSIM score typically falls within the range of (−1, 1) [32], where a higher score signifies greater similarity.

**FIGURE 2 |:**
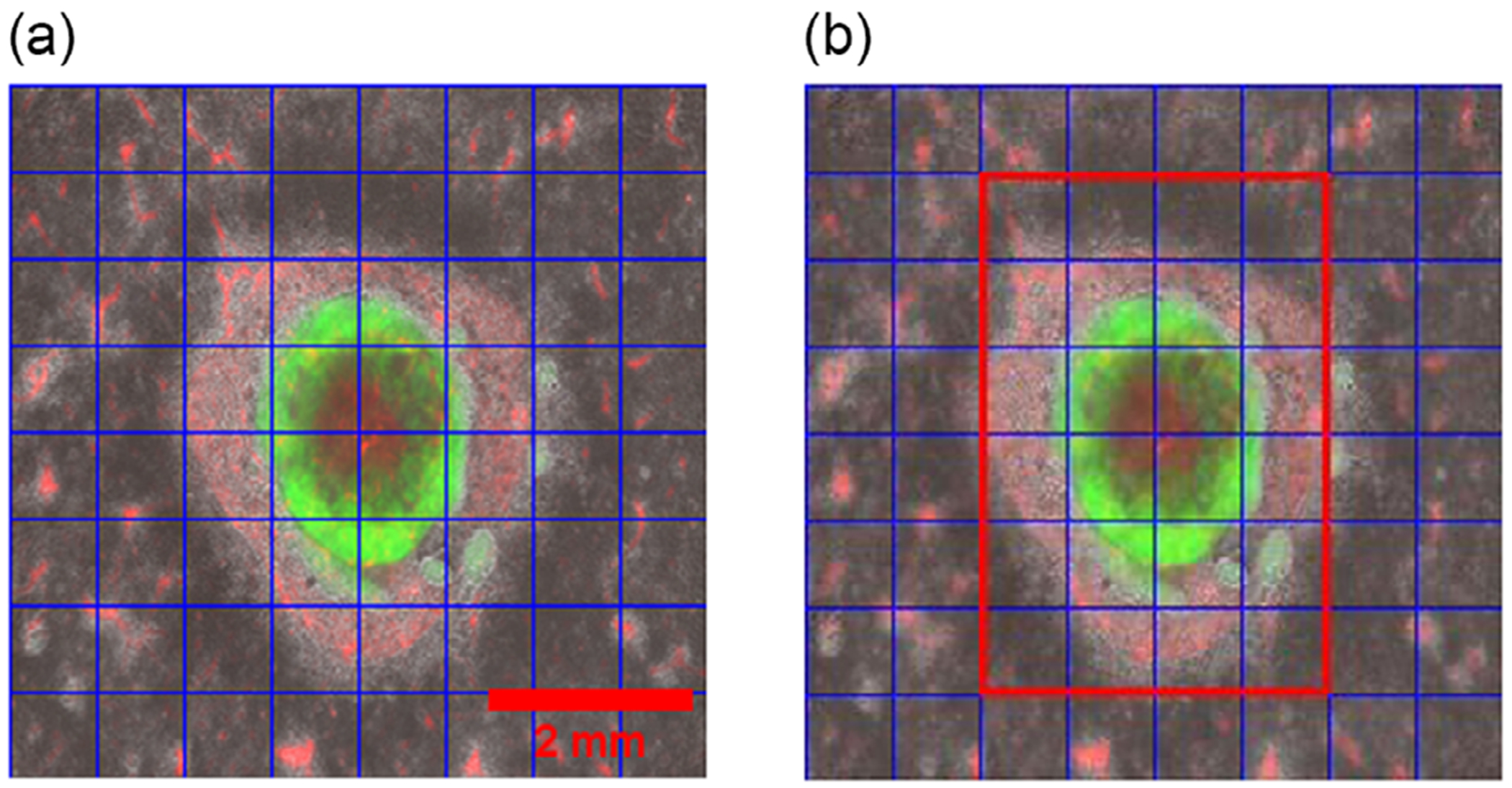
(a) Image divided into 8 × 8 grid of small patches, and (b) highlighted in red is the ROI, which is given more weightage for histogram comparison. The image size is 256 × 256, which breaks into a 16 × 16 grid with multiple patches of size at 32 × 32 pixels. Scale bar: 2000 μm.

PSNR and SSIM are widely used metrics in evaluating Image colorization tasks. Still, they are not exactly appropriate for the problem because PSNR is designed to identify the quality of the compressed image with the original image. Similarly, SSIM primarily focuses on structural similarity rather than color which is the main part of image colorization. So, the WPH was also tested to compare the similarity of generated colors. With regular histogram comparison, valuable spatial information of the color is lost, so in our approach, the images were divided into a 16 × 16 grid (Figure 2a) to have multiple small patches of the image and compare these small patches individually to the corresponding patch from the ground truth. This patch histogram comparison increased the spatial information of the pixel’s value.

As patch histogram comparison by WPH increases spatial color information; therefore, reducing the patch size to the smallest possible value may produce the best results. The smallest size possible to compare is 1 × 1 pixels, which leads to a pixel-to-pixel comparison of the images and it would be highly sensitive to noise and unreliable. So, the optimized balance between the patch size and the number of bins in the histogram comparison was tested and validated, and a patch size of 32 × 32 pixels and 32 bins was found ideal for histogram comparison in CO images. Since the most of the COs were centered in the image, the region of interest (ROI) focused on the center of CO images provided enhanced significance in color comparison without the excessive background. The weightage for the patches inside the ROI in Figure 2b was increased by 50% to give more importance to the colors in the organoids.

## 5 | Comparison and Quantification of Prediction Image of Each Cell Type in COs

Our recently published organoid image preprocessing and analysis platform, Organalysis [7], was utilized to measure and quantify the organoid area, percentage of image covered by organoid, total intensity of organoid, and total intensity of organoid-by-organoid area from the colorized images and paired ground truth of each organoid with the following calculations:

Organoid area: total pixel numbers of fluorescence per cell type in each organoid

$$\text{Percentage of Organoid Coverage}=\frac{\text{Total Area of Organoid}}{\text{Total Area of Image}}$$

$$\text{Total Intensity of Organoid by Organoid Area}=\frac{\text{Total Intensity of Organoid}}{\text{Pixel Area of Organoid}}$$

$$\text{Total Intensity of Organoid by Total Image Area}=\frac{\text{Total Intensity of Organoid}}{\text{Total Area of Image}}$$

$$\text{Difference between generated image and ground truth}=\frac{\text{Measurement of generated image}-\text{measurement of ground truth}}{\text{Measurement of ground truth}}$$

## 6 | Results

### 6.1 | Training Outcome and Optimization

First, three U-Net-based models were designed for comparing and optimizing the image colorization of hPSC-derived COs: Model 1, U-Net generator only; Model 2, U-Net generator with CBAM; Model 3, U-Net with CBAM and generator iteration. The generator iteration employed the architecture in Model 2 was trained twice in an epoch. To make the generator stronger in comparison to the discriminator, it was trained multiple times to produce more realistic colors.

**FIGURE 3 |:**
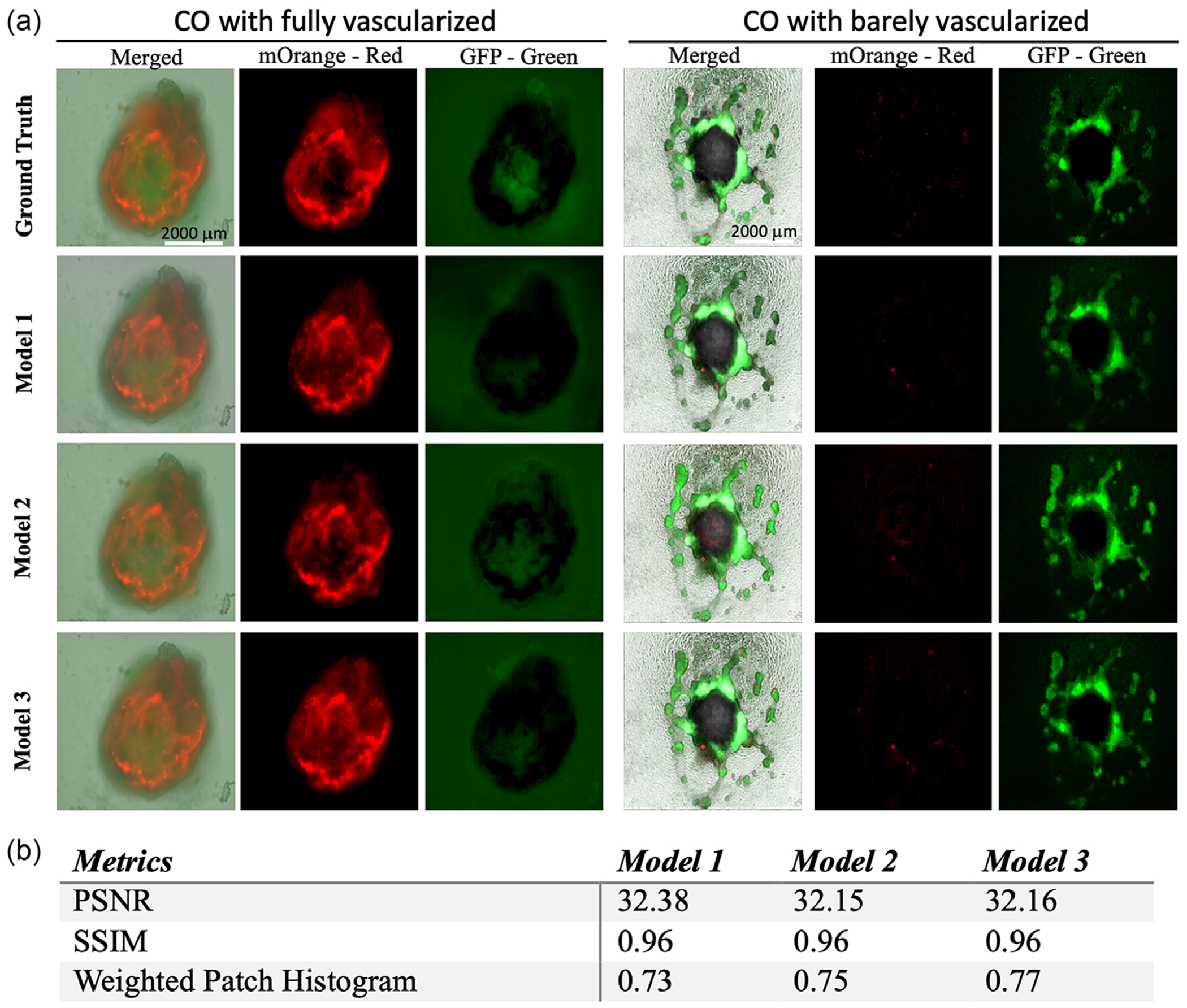
(a) Images of representative COs from ground truth and predicted images generated by Model 1, Model 2, and Model 3, respectively. COs for colorization were not included in the training dataset but from the same batch of CO differentiation. Scale bar: 2000 μm. (b) Evaluation scores by three different evaluation metrics.

After three models were trained efficiently with the training dataset of paired phase contrast and fluorescence images from the same organoids, we applied the three models for predicting the CO images in merged phase contrast and fluorescence of green and red, as shown in Figure 3a based on the phase contrast images of COs, including both fully and barely vascularized COs. Those organoids used for image prediction and fluorescence colorization were not previously included in the training dataset but from the same batch of organoid differentiation. Three evaluation metrics, the PSNR, SSIM, and WPH were employed to quantitatively evaluate the performance of our models. Figure 3b presents the outcome of evaluation scores achieved on those metrics.

The range of PSNR is [0, ∞], where 0 represents no similarity between images and infinity is for the same images. For a comparison of lossy images, the PSNR score typically ranges from 30 to 50 where the higher the score the higher the similarity [33]. Values over 40 are usually considered to be very good and anything below 20 is unacceptable [34]. The well-established techniques achieved a PSNR score of 29.52 on the COCO-stuff dataset [28], whereas our models achieved PSNR scores of over 32. The COCO-stuff dataset platform is well known for annotating images or using textual image descriptions by comparing the predicted images to the ground truth of COCO-stuff at the pixel level. The SSIM score ranges in (−1, 1) [32]. where −1 represents no similarity and 1 represents very high similarity. Therefore, a higher score indicates higher similarity. The state-of-the-art techniques have an SSIM score of 0.94 on the COCO-stuff dataset [28], whereas our models achieved SSIM scores of 0.96. Weighted patch histogram ranges in [0,1] where 0 represents no similarity in the histograms of the images, therefore no similarity, and 1 represents full similarity in histograms, resulting in a very high similarity of the images. The similarity increases from 0.73 to 0.77 from Model 1 to Model 3.

### 6.2 | Prediction of New Batches of COs and Fine-Tuning

**FIGURE 4 |:**
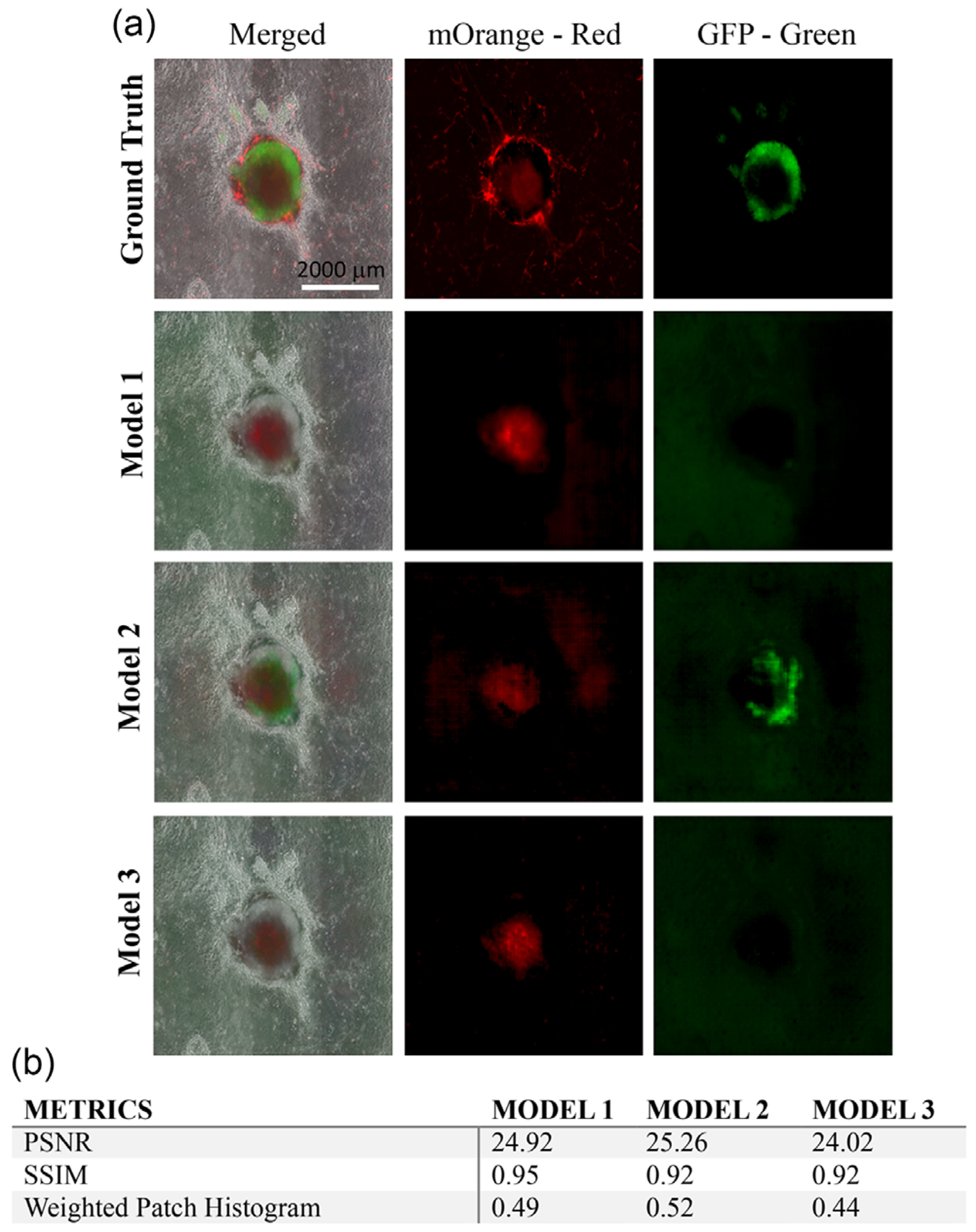
(a) Images of representative COs from ground truth and predicted images generated by Model 1, Model 2, and Model 3, respectively. COs were from different batches of CO differentiation. Scale bar: 2000 μm. (b) Evaluation score on new batches of COs.

Since all three models provided appropriate prediction results based on the similarity of the predicted image to the ground truth and evaluation metrics, they were further applied to predict the organoids from different batches of CO differentiation. As shown in Figure 4a and evaluated in Figure 4b, the predicted organoid images from Model 2 demonstrate the highest similarity in comparison with the other two models with a higher PSNR (25.26 of Model 2 vs 24.92 of Model 1 vs 24.02 of Model > 3) and weighted patch histogram (0.52 of Model 2 vs 0.49 of Model 1 vs 0.44 of Model 3). However, the results of evaluation metrics in Figure 4b indicate that the scores decrease greatly in terms of all the metrics in comparison with the prediction shown in Figure 3b. More examples of COs are further included in Figure S3 to have a more comprehensive visualization of the colorization outcome.

**FIGURE 5 |:**
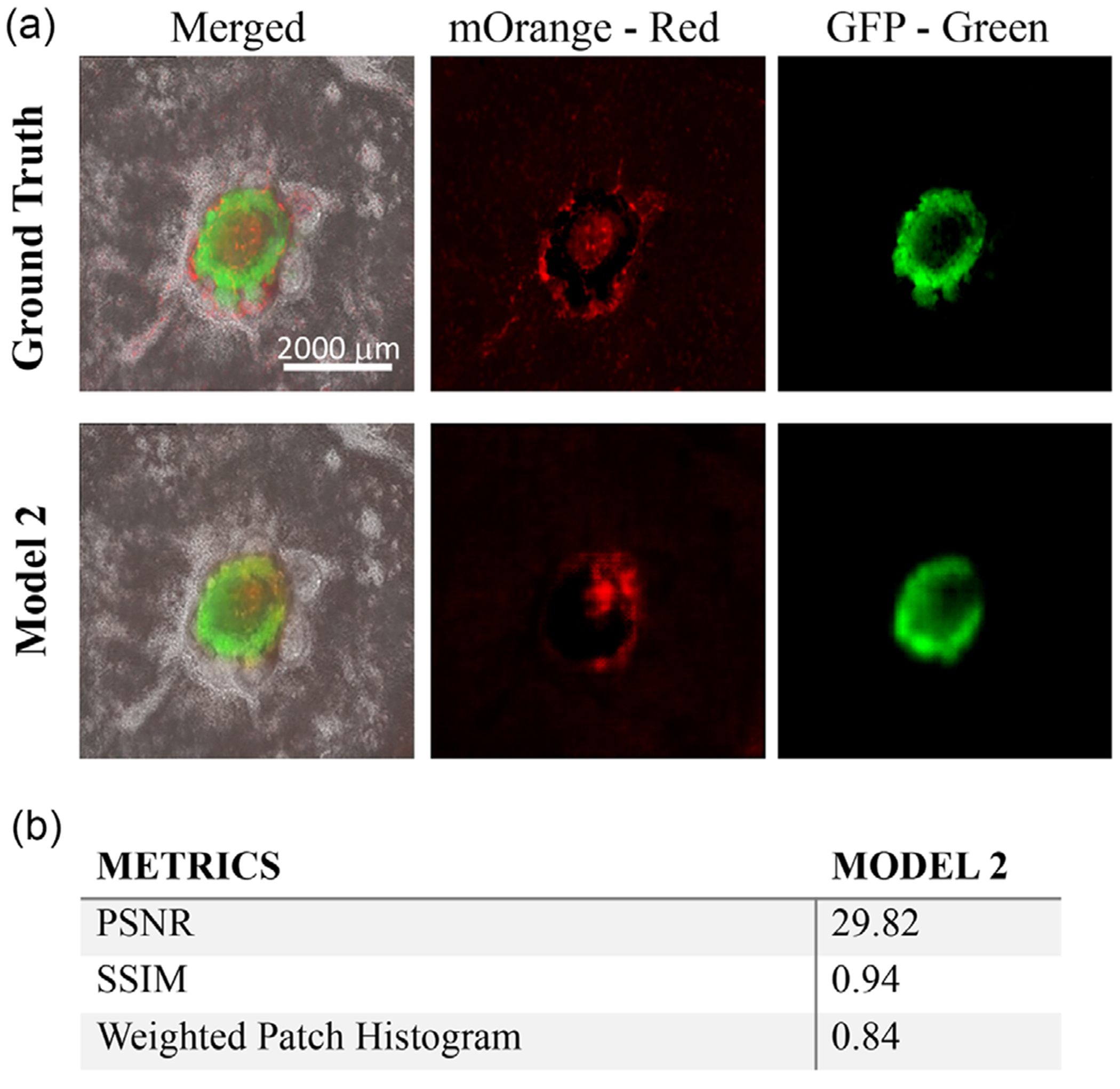
(a) Images of representative COs after fine tuning from ground truth and Model 2. COs were from different batches of CO differentiation. Scale bar: 2000 μm. (b) Evaluation score on new batches of organoids after fine-tuning.

Since Model 2 generated relatively better results than the other two models, Model 2 was further fine-tuned by retraining it with one-third of images from the new batches of CO differentiation. After Model 2 was fine tuned, Figure 5a shows the prediction results of the organoids from a new batch of differentiation. More examples of COs are further included in Figure S4 to have a more comprehensive visualization of the colorization outcome. It was found that the color generation capability of Model 2 increased after fine-tuning with improved evaluation metrics of PSNR at 29.82, SSIM at 0.94, and WPH at 0.84 (Figure 5b).

### 6.3 | Quantification and Validation of Predicted Images

To further validate the predicted organoid images, the fluorescence image of each color representing one specific type of cardiovascular cells (GFP-CM and mOrange-EC) in the COs was analyzed and quantified. The single-channeled fluorescence images were quantified by Organalysis, which is an image preprocessing software for organoid fluorescence images in high throughput, recently developed in our lab [7].

The predicted organoid images are split into individual RGB channels using Fiji [35], an ImageJ software variant, for reliability and reproducibility. These individual channels are processed using the Organalysis software. Table 1 shows the average results of Organalysis-based analysis [7] by comparing the colorized images with different measurements of COs, including organoid area, percentage of image covered by organoid, total intensity of organoid, and total intensity of organoid-by-organoid area for 70 organoids that were used for the prediction of the same batch of organoid differentiation as shown in Figure 3. The percentage of difference derived from the comparison between the generated fluorescence and the ground truth of the same organoids (difference%). If the difference% is lower than 25%, blue blocks are highlighted in Table 1. If the difference% is larger than 25%, yellow blocks are highlighted in Table 1. Accordingly, the fluorescence information from the GFP and mOrange channels generated by Model 1 is close to the ground truth. The fluorescence measurements on the GFP and mOrange channels generated by Model 2 were close to the ground truth with difference% in lower or close to 25% in organoid area and percentage of image covered by organoid. Model 3 performed well, also showing a low difference% in all the measurements of organoid fluorescence image quantification.

Moreover, the cGAN-generated fluorescence information of an additional 25 COs from a new batch of CO differentiation, as shown in Figure 4a and S3, were further extracted and quantified by Organalysis. In Table 2, nearly all the models generated the fluorescence information for all three channels at a high difference%, which aligns with the results of evaluation metrics in Figure 4b. After fine-tuning Model 2, Table 3 shows the quantification results by Organalysis of representative images in Figure 5 and S4. The images of GFP-labeled CMs in the COs generated by the fine-tuned Model 2 are very close to the ground truth with less than a 16% difference to the ground truth in both organoid area and intensity in green fluorescence. The mOrange-labeled ECs generated by the GAN model are also close to the ground truth regarding the organoid area. However, the total intensity of generated mOrange fluorescence is over 30% difference in comparison with the ground truth.

## 7 | Discussion

hPSC-derived COs are the most emerging in vitro human heart model, which has been used from basic developmental biology to translational drug discovery and regenerative medicine; however, how to characterize COs in high efficiency and efficacy at examining cardiovascular cell type and corresponding quantifications without additional fluorescence immunostaining and imaging has not been achieved yet. This study filled this gap by introducing a novel strategy for fluorescently colorizing COs from phase contrast microscopic images by utilizing cGANs and CBAM. The findings of the study illustrate the efficiency of this framework in capturing fluorescence intricacies of the cardiovascular cells (CMs and ECs) in the vascularized COs.

To better evaluate the prediction outcomes from the algorithms of cGANs + CBAM, three different evaluation metrics were applied with varied emphasis and focus on image recognition and comparison. For example, the WPH was included as a new metric to highlight the efficacy of our approach in preserving biological details compared to traditional metrics like PSNR and SSIM. Typically, the images generated with evaluation scores of PSNR over 30, SSIM over 0.92, and a WPH score over 0.75 are the most accurate and similar to the ground truth.

Initially, the prediction of fluorescence images within the same batch of organoid differentiation was highly accurate, especially by integrating the CBAM into the conditional GAN framework of Model 2, which captured salient features in phase contrast images of COs. This attention mechanism enhances the quality and fidelity of the generated colorizations by directing the model’s focus toward critical regions within the image and generating realistic and accurate colorizations of grayscale organoid images [36]. To further test the prediction outcome of organoids differentiated from different batches, we included additional organoids from the other two new batches of organoid differentiation. However, the prediction accuracy was greatly reduced in PSNR and WPH. The COs in the new batch of differentiation, although representing the same type of organoid differentiated with the same protocol, still exhibit subtle variations in image presentations not adequately captured during the initial training. These variations might include subtle differences in biological variability between CO batches (e.g., slight differences in morphology or fluorescence intensity distribution), and/or other factors that introduce “new patterns” not present in the original training process. The model before fine-tuning has not learned the features necessary to accurately colorize images with these new patterns. Fine-tuning, therefore, acts as a crucial adaptation step, allowing the model to learn these dataset-specific features and adjust its parameters accordingly. To address this problem and bolster the prediction accuracy of organoids from the different batches of differentiation, we did fine-tuning by incorporating one third of organoid images from the new batches of differentiation into the training dataset. This step of fine turning did improve the prediction outcome with higher evaluation metrics. Therefore, we suggest to incorporate a broader spectrum of CO batches in the training dataset to enable the model to capture and learn the subtle differences across different CO batches and generate robust and accurate fluorescence.

Finally, to meet the general need for organoid characterization by image quantification, we conducted the fluorescence image analysis and comparison between prediction and ground truth. We adapted the most common measurement of organoid images focusing on each cardiovascular-specific cell type (CMs and ECs): organoid area, percentage of image covered by organoid, total intensity of organoid, and total intensity of organoid-by-organoid area. The percentage of differences (difference%) in organoid area, percentage of image covered by organoid, total intensity of organoid, and total intensity of organoid-by-organoid area are all lower than 25% in the prediction of the same batches of organoids in G (GFP-CMs) and R (mOrange-ECs). Through the optimization of fine tuning, the difference% in GFP and mOrange fluorescences becomes lower than 10% in the organoid area and percentage of image covered by organoid with significant improvement in the fluorescence colorization of hPSC-derived COs. However, the prediction of fluorescence intensity-related measurements needs further improvements due to the variation of microscopic imaging from different batches of CO differentiation even using the same imaging setup and parameters.

### 7.1 | Limitations and Future Works

Although the established cGAN + CBAM algorithm has achieved satisfied predictions of CO fluorescence images from the corresponding phase contrast images, a few limitations still need to be further addressed to improve the prediction accuracy with additional functions. For example, the prediction of mOrange-EC fluorescence’s total intensity could be further enhanced. To overcome this limitation, we will increase the dataset size with more images at varied sample categories, such as including the COs with broader variation and defined ratios of each fluorescence through controlled organoid differentiation. Also, we will consider employing ensemble learning techniques, where multiple models are trained, and their predictions are combined to improve overall accuracy and robustness. As supported by the results of fine-tuning, the prediction accuracy was enhanced significantly; however, how to achieve a promising prediction outcome without fine turning has not been achieved yet. Prospectively achieving this level of comprehensive data representation is exceptionally challenging in practice. We will include extensive experimentation and data collection with more resources, time, and all possible variations from features and patterns of COs. Since increasing the dataset size could introduce the dataset diversity, we will carefully balance the dataset diversity with training efficiency. We will adapt possible strategies to optimize this balance, such as exploring active learning to intelligently select the most informative samples for inclusion in the training set and employing efficient network architectures. We will also try incorporating the progressive GAN [37] technique in our training approach to enhance training stability and capture intricate details of COs to skip the step of fine tuning and still achieve high accuracy of fluorescence colorization. Accordingly, the predicted image quantification related to fluorescence intensity measurement will be improved further for the organoids from a new batch of differentiation. In future work, we will also plan to systematically evaluate the model on COs derived from multiple hPSC lines, explore transfer learning where a model pretrained on one line is fine-tuned on data from others, and investigate whether the learned features are generalizable across cell lines or if line-specific features need to be incorporated. The cGAN used here was designed with flexibility, enabling the adjustment of resolution for both input and output images. The generator and discriminator within the cGAN architecture maintain consistent spatial dimensions, ensuring precise dimensional alignment between input and output images. In future research, we will aim to enhance computational capabilities by employing higher-resolution images (e.g., 1024 pixel × 1024 pixel) to achieve more accurate predictions of intricate cellular structures, such as vascular networks and cardiac complexities. Further improvements in cell recognition and classification accuracy are certainly possible, for example, higher resolution imaging by exploring the use of higher magnification objectives or super-resolution microscopy to improve the resolution of individual cells, explicit overlap handling through segmentation techniques or modified loss functions that penalize misidentification or overlap in dense regions, and incorporating additional markers, potentially adding additional cell type-specific markers to further enhance discrimination. Another limitation of the current study is that only epi-fluorescence images were included in the training dataset. In consideration of the three-dimensional (3D) structure of COs, the confocal fluorescence microscopic imaging with a 3D image stack will be considered to predict the 3D structure of COs with cell type-specific expressions and networks. Finally, the prediction of CO differentiated from more hPSC lines could be included and evaluated to extend the biomedical application of the fluorescence colorization model.

## 8 | Conclusions

In conclusion, a new Generative AI model was established to address the critical challenge of colorizing phase images of hPSC-derived COs using cGANs and CBAM. This framework has demonstrated its efficacy in capturing intricate multichannel fluorescence information within each organoid. It also enhances the interpretability and analysis of cardiovascular cell type in both images and quantification for biomedical research and applications. The cGAN model, enriched by the CBAM module, outperformed the other two models, showcasing its adaptability and effectiveness by evaluating and comparing three evaluation metrics. Notably, for optimal results on the organoid from new batches of differentiation, fine tuning the model was suggested, ensuring that accurate and faithful fluorescence information was generated. Moreover, the quantification of GFP and mOrange fluorescence information in predicted organoid images brings extensive validation of COs for broader and impactful biomedical applications, such as the prediction of cell type-specific drug cardiotoxicity, prediction of cardiovascular development, sex, race, and genetic/mutation-specific disease evaluations, if more diverse hPSC cell lines are included in the training dataset. A similar algorithm or strategy can also be applied to the brain, liver, kidney, and cancer organoids for automatic fluorescence colorization and quantification.

## Supplementary Material

Supplementary

## Figures and Tables

**TABLE 1 |:** Quantification and comparison of the individual fluorescence channels in ground truth and predicted images of COs from the same batch of differentiation.

	Metric	Organoid area		Percentage of image covered by organoid		Total intensity of organoid		Total intensity of organoid by organoid area	
		mOr-EC	GFP-CM	mOr-EC	GFP-CM	mOr-EC	GFP-CM	mOr-EC	GFP-CM
Model 1	Average of Model 1	5,287.3	12,346.3	8.1	18.8	191,929.2	647,300.4	29.8	56.9
	Average ground truth	4,813.0	12,176.6	7.3	18.6	185,273.8	674,256.1	31.3	58.0
	Difference%^a^	9.9	1.4	9.9	1.4	3.6	4.0	4.9	2.0
Model 2	Average of Model 2	6,057.4	14,409.4	9.2	22.0	197,167.2	704,817.9	28.4	59.6
	Average ground truth	4,813.0	12,176.6	7.3	18.6	185,273.8	674,256.1	31.3	58.0
	Difference%^a^	25.9	18.3	25.9	18.3	6.4	4.5	9.5	2.7
Model 3	Average of Model 3	5,615.3	13,963.3	8.6	21.3	190,031.7	675,796.4	28.5	58.7
	Average ground truth	4,813.0	12,176.6	7.3	18.6	185,273.8	674,256.1	31.3	58.0
	Difference%^a^	16.7	14.7	16.7	14.7	2.6	0.2	9.1	1.2

a Blue blocks show low difference% less than 25% and yellow blocks show high difference% more than 25%.

**TABLE 2 |:** Quantification and comparison of the individual fluorescence channels in the ground truth and predicted images of COs from a new batch of differentiation.

	Metric	Organoid area		Percentage of image covered by organoid		Total intensity of organoid		Total intensity of organoid by organoid area	
		mOr-EC	GFP-CM	mOr-EC	GFP-CM	mOr-EC	GFP-CM	mOr-EC	GFP-CM
Model 1	Average of Model 1	3,909.8	25,471.5	6.0	38.9	29,111.6	381,391.7	14.7	20.3
	Average ground truth	5,164.9	3,258.8	7.9	5.0	145,953.5	212,736.2	29.3	65.4
	Difference%^a^	24.3	681.6	24.3	681.6	80.1	79.3	49.8	68.9
Model 2	Average of Model 2	4,658.3	12,125.7	7.1	18.5	50,595.1	232,752.4	12.1	37.5
	Average ground truth	5,164.9	3,258.8	7.9	5.0	145,953.5	212,736.2	29.3	65.4
	Difference%^a^	9.8	272.1	9.8	272.1	65.3	9.4	58.8	42.7
Model 3	Average of Model 3	1,333.9	39,878.6	2.0	60.8	22,187.4	570,512.7	16.4	13.8
	Average ground truth	5,164.9	3,258.8	7.9	5.0	145,953.5	212,736.2	29.3	65.4
	Difference%^a^	74.2	1123.7	74.2	1123.7	84.8	168.2	44.0	78.9

a Blue blocks show low difference% less than 25% and yellow blocks show high difference% more than 25%.

**TABLE 3 |:** Quantification and comparison of the individual fluorescence channels in the ground truth and predicted images of COs from a new batch of differentiation upon fine tuning.

Metric	Organoid area		Percentage of image covered by organoid		Total intensity of organoid		Total intensity of organoid by organoid area	
	mOr-EC	GFP-CM	mOr-EC	GFP-CM	mOr-EC	GFP-CM	mOr-EC	GFP-CM
Average of Model 2	4,232.5	2,946.3	6.5	4.5	56,174.0	121,931.8	15.6	40.4
Average ground truth	4,606.7	3,178.5	7.0	4.9	103,742.0	166,253.0	22.2	52.9
Difference%^a^	8.1	7.3	8.1	7.3	45.9	26.7	29.7	23.7

a Blue blocks show low difference% less than 25% and yellow blocks show high difference% more than 25%.

## Data Availability

The data that support the findings of this study are available from the corresponding author upon reasonable request.
