# Supplementary figures and images for: Generative Ai for Cardiovascular Cell Type-Specific Fluorescence Colorization of Live-Cell hPSC-Derived Cardiac Organoids
Source: Adv Intell Discov. Author manuscript; Available in PMC 2025 Aug 25. (PMC12373119; doi:10.1002/aidi.202400041)

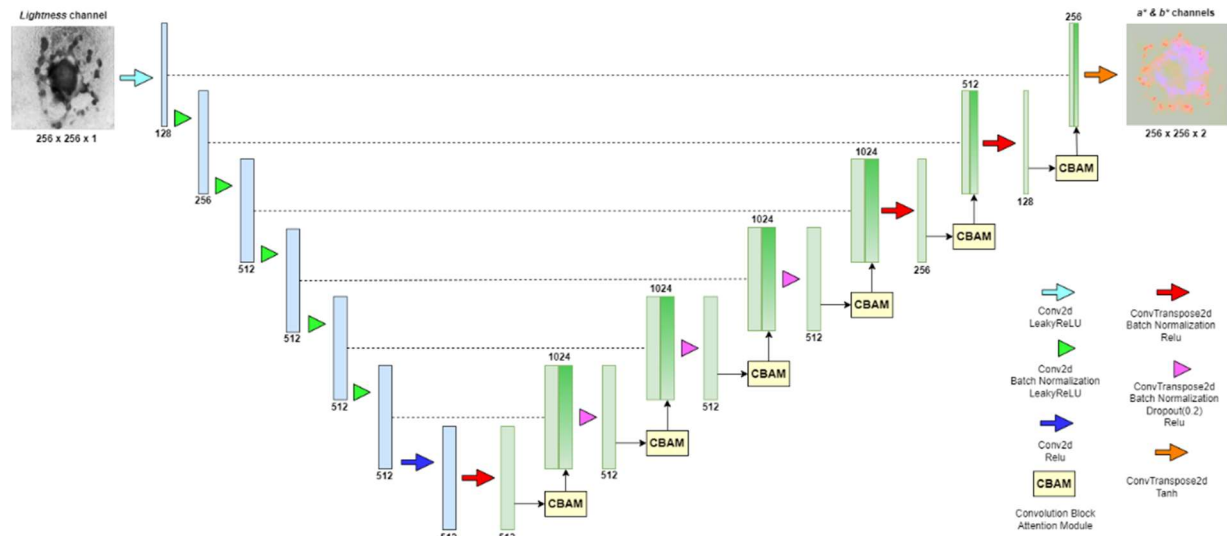

Supplementary Figure 2

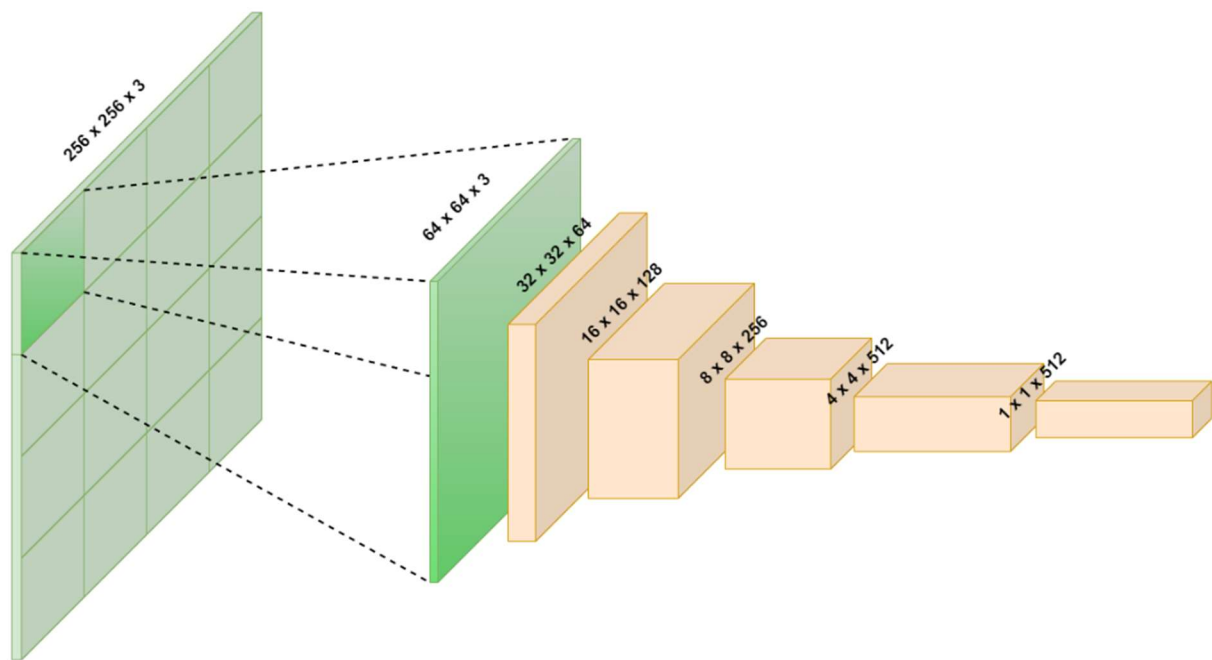

Supplementary Figure 3

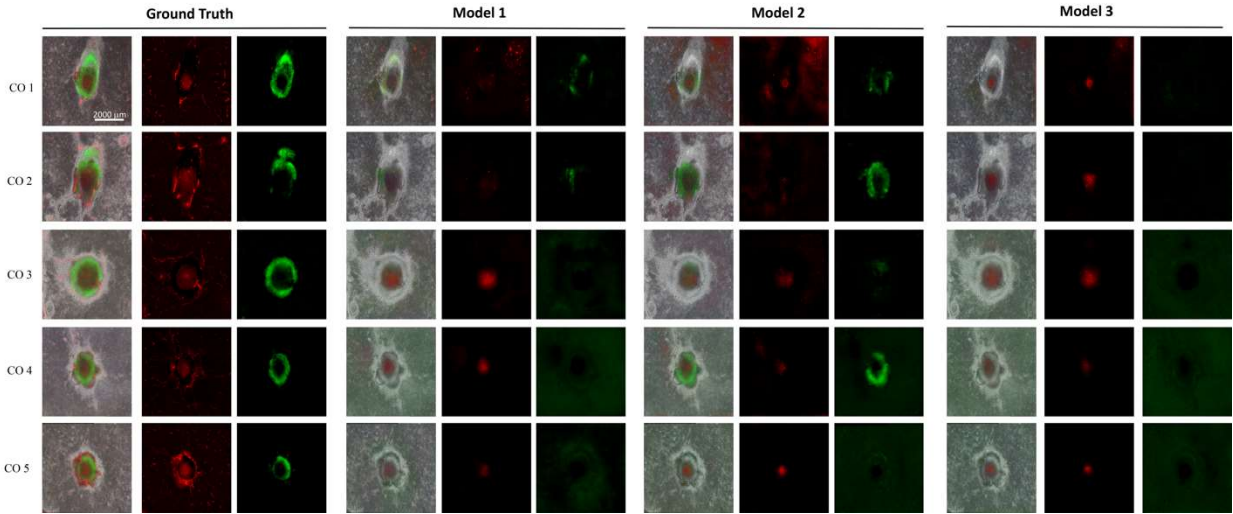

Supplementary Figure 4

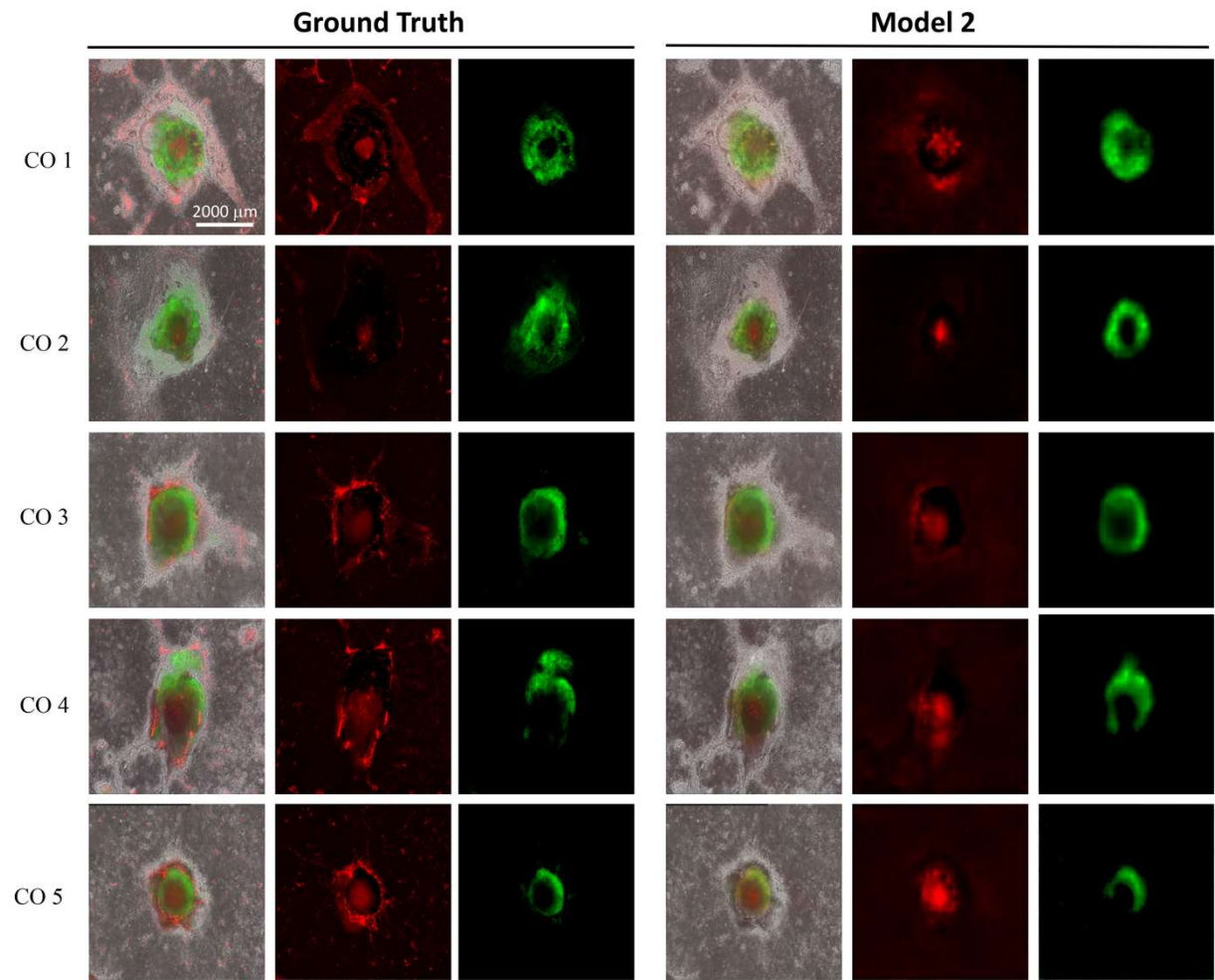

Supplement: Supplementary [file NIHMS2079463-supplement-Supplementary.pdf]
